# Continued Low Efficacy of Artemether-Lumefantrine in Angola in 2019

**DOI:** 10.1128/AAC.01949-20

**Published:** 2021-01-20

**Authors:** Pedro Rafael Dimbu, Roberta Horth, Ana Luísa M. Cândido, Carolina Miguel Ferreira, Felismina Caquece, Luzala Elisabeth Armando Garcia, Kialanda André, Garcia Pembele, Domingos Jandondo, Belmira José Bondo, Benjamin Nieto Andrade, Sarah Labuda, Gabriel Ponce de León, Julia Kelley, Dhruviben Patel, Samaly S. Svigel, Eldin Talundzic, Naomi Lucchi, Joana F. M. Morais, Filomeno Fortes, José Franco Martins, Mateusz M. Pluciński

**Affiliations:** a National Malaria Control Program, Ministry of Health, Luanda, Angola; b Epidemic Intelligence Service, U.S. Centers for Disease Control and Prevention, Atlanta, Georgia, USA; c National Institute of Health Research, Ministry of Health, Luanda, Angola; d Population Services International Angola, Luanda, Angola; e Field Epidemiology Training Program, Ministry of Health, Luanda, Angola; f Malaria Branch, Centers for Disease Control and Prevention, Atlanta, Georgia, USA; g U.S. President’s Malaria Initiative, Centers for Disease Control and Prevention, Luanda, Angola; h U.S. President’s Malaria Initiative, Centers for Disease Control and Prevention, Atlanta, Georgia, USA; i Institute of Hygiene and Tropical Medicine, Nova University of Lisbon, Lisbon, Portugal

**Keywords:** malaria, resistance, molecular markers

## Abstract

Biennial therapeutic efficacy monitoring is a crucial activity for ensuring the efficacy of currently used artemisinin-based combination therapy in Angola. Children with acute uncomplicated Plasmodium falciparum infection in sentinel sites in the Benguela, Zaire, and Lunda Sul Provinces were treated with artemether-lumefantrine (AL) or artesunate-amodiaquine (ASAQ) and monitored for 28 days to assess clinical and parasitological responses. Molecular correction was performed using seven microsatellite markers. Samples from treatment failures were genotyped for the *pfk13*, *pfcrt*, and *pfmdr1* genes. Day 3 clearance rates were ≥95% in all arms. Uncorrected day 28 Kaplan-Meier efficacy estimates ranged from 84.2 to 90.1% for the AL arms and 84.7 to 100% for the ASAQ arms. Corrected day 28 estimates were 87.6% (95% confidence interval [CI], 81 to 95%) for the AL arm in Lunda Sul, 92.2% (95% CI, 87 to 98%) for AL in Zaire, 95.6% (95% CI, 91 to 100%) for ASAQ in Zaire, 98.4% (95% CI, 96 to 100%) for AL in Benguela, and 100% for ASAQ in Benguela and Lunda Sul. All 103 analyzed samples had wild-type *pfk13* sequences. The 76T *pfcrt* allele was found in most (92%; 11/12) ASAQ late-failure samples but in only 16% (4/25) of AL failure samples. The N86 *pfmdr1* allele was found in 97% (34/35) of treatment failures. The AL efficacy in Lunda Sul was below the 90% World Health Organization threshold, the third time in four rounds that this threshold was crossed for an AL arm in Angola. In contrast, the observed ASAQ efficacy has not been below 95% to date in Angola, including this latest round.

## Abstract (precis)

Biennial therapeutic efficacy monitoring is a crucial activity for ensuring the efficacy of currently used artemisinin-based combination therapy in Angola. Children with acute uncomplicated Plasmodium falciparum infection in sentinel sites in the Benguela, Zaire, and Lunda Sul Provinces were treated with artemether-lumefantrine (AL) or artesunate-amodiaquine (ASAQ) and monitored for 28 days to assess clinical and parasitological responses. Molecular correction was performed using seven microsatellite markers.

## INTRODUCTION

Malaria is a major public health concern in Angola, with over 7 million cases registered in 2019. Most cases are caused by Plasmodium falciparum, and the cornerstone of malaria case management is prompt diagnosis and treatment with artemisinin-based combination therapy (ACT). Three ACTs are used as first-line treatments in the public health sector in Angola: artemether-lumefantrine (AL), artesunate-amodiaquine (ASAQ), and dihydroartemisinin-piperaquine (DP).

Antimalarial resistance has been a continuous threat to malaria control throughout the globe. The emergence of artemisinin resistance in Southeast Asia (1) presents the risk of its spread to sub-Saharan Africa, mirroring the emergence and spread of chloroquine and sulfadoxine-pyrimethamine resistance (2, 3). In addition, there have been recent reports of the independent emergence of artemisinin resistance in Rwanda (4). To mitigate these threats, the World Health Organization (WHO) recommends periodic reassessment of the efficacy of currently used antimalarials in countries where the disease is endemic (5).

While initial pre-ACT-era trials in Angola showed 100% efficacies of AL and ASAQ (6), routine biennial therapeutic efficacy monitoring in Angola’s three sentinel sites has shown signs of AL efficacy hovering around the crucial WHO 90% threshold in the Zaire Province site (7–9) (Table 1). In contrast, the lowest observed efficacies for ASAQ and DP in any site have been 93.3% and 98.8%, respectively.

**TABLE 1:** Kaplan-Meier estimates of day 28 (AL and ASAQ) and day 42 (DP) corrected efficacies in previous rounds of therapeutic monitoring in Angolan sentinel sites from 2013 to 2017

Yr	Reference	Efficacy (%)^*c*^										
		Zaire			Benguela			Lunda Sul			Uíge^*a*^	
		AL	ASAQ	DP	AL	ASAQ	DP	AL	ASAQ	DP	AL	DP
2013	7	89.6		100^*b*^							97.4	100^*b*^
2015	8	88.1		98.8	96.3	99.9			100	100		
2017	9	95.5	93.3			100	100	96.5		100		

a Discontinued as a sentinel site after 2013.

b Day 28 only.

c AL, artemether-lumefantrine; ASAQ, artesunate-amodiaquine; DP, dihydroartemisinin-piperaquine.

Characterization of molecular markers of resistance in samples collected during therapeutic efficacy monitoring complements and facilitates the interpretation of the clinical data. To date, as in most sub-Saharan settings, no *pfk13* mutations associated with artemisinin resistance have been detected in Angola either as part of therapeutic efficacy monitoring or in separate molecular surveys (7–12). Molecular surveys in Angola have shown a predominance of the K76T *pfcrt* mutation (10), conclusively linked to chloroquine resistance and also potentially associated with amodiaquine resistance but lumefantrine sensitivity (13). Consistent with this putative relationship, all ASAQ treatment failure samples in the 2017 round of Angolan monitoring had the 76T mutated *pfcrt* allele, whereas 88% of AL treatment failures had the K76 wild-type allele. Analysis of AL treatment failure samples from the 2013, 2015, and 2017 rounds found a predominance of the N86 *pfmdr1* allele previously associated with decreased sensitivity to lumefantrine (13). In addition, in the 2015 study, all pretreatment samples regardless of treatment outcome were sequenced for *pfmdr1*, and a large majority (86%) had the N86 allele present (11).

Here, the results of the fourth round of therapeutic efficacy monitoring in Angola’s three fixed sentinel sites are reported.

## RESULTS

Across all study arms, 3,616 children were screened, and 610 (16.8%) entered the study (Table 2). Twenty (3.3%) were lost to follow-up, and a further 11 (1.8%) were excluded. Rates of loss to follow-up were ≤5% across all arms. The median age varied between 2.8 years in the Zaire arms and 7.5 years in the Benguela ASAQ arm (Table 2). The median parasite density and hemoglobin level at enrollment varied by study arm between 19,114 and 44,467 parasites/μl and 10.0 to 10.8 g/dl, respectively.

**TABLE 2:** Number of participants screened, enrolled, and finishing follow-up and characteristics at baseline as part of therapeutic efficacy monitoring in Angola in 2019

Parameter	Value for arm					
	Benguela		Zaire		Lunda Sul	
	AL	ASAQ	AL	ASAQ	AL	ASAQ
No. of participants screened	682	806	475	450	468	735
No. enrolled	105	100	100	100	100	105
No. lost to follow-up (%)	2 (2)	1 (1)	3 (3)	5 (5)	4 (4)	5 (5)
No. excluded (%)	2 (2)	1 (1)	2 (2)	4 (4)	1 (1)	1 (1)
No. reaching study endpoint (%)	101 (96)	98 (98)	95 (95)	91 (91)	95 (95)	99 (94)

Participant characteristics at baseline						
Median age (yrs) (range)	6.5 (0.9–12)	7.5 (0.5–12)	2.8 (0.5–5)	2.8 (0.5–5)	3 (0.8–5)	3.4 (0.7–5)
Median wt (kg) (range)	18 (9–32)	20 (7–42)	12 (6–21)	12 (6–18)	12 (7–18)	13 (7–23)
% female participants	43	47	54	53	48	47
Median day 0 parasitemia (no. of parasites/μl) (range)	19,114 (1,030–97,313)	35,847 (1,048–95,067)	37,352 (2,284–198,595)	44,467 (2,087–196,176)	26,159 (2,120–193,346)	27,066 (2,190–186,419)
Median day 0 hemoglobin level (g/dl) (range)	10.7 (8.1–13.9)	10.4 (8.1–14.3)	10.6 (8–13.5)	10.8 (8.1–13.4)	10 (8.1–13.3)	10.4 (8.2–13.5)
Start mo-end mo	Mar-May	Mar-Apr	Mar-Apr	Mar-May	Mar-May	Mar-Jul

The rate of day 2 slide negativity was 68% (95% confidence interval [CI], 58 to 77%) in the AL arm in Lunda Sul but was above 80% in all other arms (Table 3). Day 3 slide negativity rates ranged between 95% and 100%. There were 1 early treatment failure and 53 cases of recurrent parasitemia (Table 4). Of the 53 cases of recurrent parasitemia, 24 had a probability of recrudescence of >0.5 (Table 4; see also Tables S1 and S2 in the supplemental material).

**TABLE 3:** Proportions of slides negative for asexual malaria parasites on days 2 and 3 following antimalarial treatment during therapeutic efficacy monitoring in Angola in 2019

Day	Proportion (%) of negative slides (95% CI)					
	Benguela		Zaire		Lunda Sul	
	AL	ASAQ	AL	ASAQ	AL	ASAQ
2	92 (85–96)	91 (83–96)	84 (75–90)	96 (89–99)	68 (58–77)	91 (84–96)
3	99 (94–100)	100 (95–100)	99 (94–100)	100 (95–100)	95 (88–98)	99 (94–100)

**TABLE 4:** Treatment outcomes for participants finishing follow-up as part of therapeutic efficacy monitoring in Angola in 2019

Outcome	No. of participants (%)					
	Benguela		Zaire		Lunda Sul	
	AL (*n *= 101)	ASAQ (*n* = 98)	AL (*n* = 95)	ASAQ (*n* = 91)	AL (*n *= 95)	ASAQ (*n* = 99)
Treatment failure	10 (10)	0	16 (17)	14 (15)	14 (15)	0
Early treatment failure	0	0	0	1 (1)	0	0
Recurrent parasitemia	10 (10)	0	16 (17)	13 (14)	14 (15)	0
Recrudescence	1 (1)	0	8 (8)	3 (3)	12 (13)	0
Day 14	1 (1)	0	1 (1)	0	2 (2)	0
Day 21	0	0	5 (5)	1 (1)	9 (9)	0
Day 28	0	0	2 (2)	2 (2)	1 (1)	0
Reinfection	9 (9)	0	8 (8)	10 (11)	2 (2)	0
Day 14	0	0	1 (1)	1 (1)	2 (2)	0
Day 21	3 (3)	0	2 (2)	5 (5)	0	0
Day 28	6 (6)	0	5 (5)	4 (4)	0	0

Adequate clinical and parasitological response	91 (90)	98 (100)	79 (83)	77 (85)	81 (85)	99 (100)

The uncorrected Kaplan-Meier estimates of the day 28 efficacy were 84.2% (95% CI, 77 to 92%) for the AL arm in Zaire, 84.7% (78 to 92%) for ASAQ in Zaire, 85.3% (79 to 93%) for AL in Lunda Sul, 90.1% (85 to 96%) for AL in Benguela, and 100% for the ASAQ arms in Benguela and Lunda Sul (Table 5). After molecular correction, the Kaplan-Meier estimates were 87.6% (95% CI, 81 to 95%) for the AL arm in Lunda Sul, 92.2% (87 to 98%) for AL in Zaire, 95.6% (91 to 100%) for ASAQ in Zaire, 98.4% (96 to 100%) for AL in Benguela, and 100% for ASAQ in Benguela and Lunda Sul.

**TABLE 5:** Efficacy of first-line antimalarials in three therapeutic efficacy monitoring sites in Angola in 2019

Type of estimate	% efficacy (95% CI)					
	Benguela		Zaire		Lunda Sul	
	AL	ASAQ	AL	ASAQ	AL	ASAQ
Uncorrected Kaplan-Meier estimate, day 28	90.1 (85–96)	100^*a*^	84.2 (77–92)	84.7 (78–92)	85.3 (79–93)	100^*a*^
PCR-corrected Kaplan-Meier estimate, day 28	98.4 (96–100)	100^*a*^	92.2 (87–98)	95.6 (91–100)	87.6 (81–95)	100^*a*^

a Confidence intervals are undefined.

All 103 sequenced samples from patients with early treatment failure at day 0, and day 0 and day-of-failure samples from participants with recurrent parasitemia, were wild type for *pfk13* (Table 6). Nearly all (92%; 11/12) ASAQ day-of-failure samples from cases of recurrent parasitemia had the 76T *pfcrt* allele. In contrast, only 16% (4/25) of AL day-of-failure samples had this allele. All (21/21) AL and most (93%; 13/14) ASAQ day-of-failure samples had the N86 *pfmdr1* allele.

**TABLE 6:** Molecular markers of resistance for treatment failures observed during therapeutic efficacy monitoring in Angola, stratifying by treatment, in 2019^*a*^

Marker	No. of positive samples/total no. of samples tested (%)								
	AL				ASAQ				
	Reinf day 0 (*n* = 19)	Reinf day of failure (*n* = 19)	Recr day 0 (*n *= 21)	Recr day of failure (*n* = 21)	ETF (*n* = 1)	Reinf day 0 (*n* = 10)	Reinf day of failure (*n* = 10)	Recr day 0 (*n* = 3)	Recr day of failure (*n* = 3)
*pfk13*									
Wild type	19/19 (100)	18/18 (100)	21/21 (100)	18/18 (100)	1/1 (100)	10/10 (100)	10/10 (100)	3/3 (100)	3/3 (100)

*pfcrt*									
C72	18/18 (100)	15/15 (100)	19/19 (100)	10/10 (100)	1/1 (100)	10/10 (100)	8/8 (100)	3/3 (100)	2/2 (100)
72S	0/18 (0)	0/15 (0)	0/19 (0)	0/10 (0)	0/1 (0)	0/10 (0)	0/8 (0)	0/3 (0)	0/2 (0)
M74	13/18 (72)	11/15 (73)	15/19 (79)	10/10 (100)	1/1 (100)	6/10 (60)	1/9 (11)	2/3 (67)	0/2 (0)
74I	5/18 (28)	4/15 (27)	7/19 (37)	0/10 (0)	0/1 (0)	5/10 (50)	9/9 (100)	3/3 (100)	2/2 (100)
N75	13/18 (72)	11/15 (73)	15/19 (79)	10/10 (100)	1/1 (100)	6/10 (60)	1/9 (11)	2/3 (67)	0/2 (0)
75E	5/18 (28)	4/15 (27)	7/19 (37)	0/10 (0)	0/1 (0)	5/10 (50)	9/9 (100)	2/3 (67)	2/2 (100)
K76	13/18 (72)	11/15 (73)	15/19 (79)	10/10 (100)	1/1 (100)	6/10 (60)	0/9 (0)	2/3 (67)	1/3 (33)
76T	5/18 (28)	4/15 (27)	7/19 (37)	0/10 (0)	0/1 (0)	5/10 (50)	9/9 (100)	3/3 (100)	2/3 (67)

*pfmdr1*									
N86	14/14 (100)	9/9 (100)	16/17 (94)	12/12 (100)	1/1 (100)	9/9 (100)	9/10 (90)	3/3 (100)	3/3 (100)
86Y	0/14 (0)	0/9 (0)	1/17 (6)	0/12 (0)	0/1 (0)	0/9 (0)	1/10 (10)	1/3 (33)	1/3 (33)
Y184	10/13 (77)	9/9 (100)	15/17 (88)	9/10 (90)	0/1 (0)	9/10 (90)	7/10 (70)	2/3 (67)	3/3 (100)
184F	4/13 (31)	0/9 (0)	6/17 (35)	2/10 (20)	1/1 (100)	2/10 (20)	6/10 (60)	1/3 (33)	0/3 (0)
D1246	12/12 (100)	10/10 (100)	17/17 (100)	7/7 (100)	1/1 (100)	9/10 (90)	9/10 (90)	3/3 (100)	3/3 (100)
1246Y	0/12 (0)	0/10 (0)	0/17 (0)	0/7 (0)	0/1 (0)	1/10 (10)	1/10 (10)	0/3 (0)	0/3 (0)

a ETF, early treatment failure; Reinf, reinfection; Recr, recrudescence.

## DISCUSSION

For the third time in four rounds of Angolan therapeutic efficacy monitoring, one of the AL arms was observed to have a corrected efficacy below the key 90% threshold set by the WHO. While previous rounds have shown suboptimal AL efficacy in Zaire, the AL efficacy in Zaire for this round was 92.2%. In contrast, the AL efficacy in Lunda Sul for this round was 87.6%, compared to 96.5% in 2017. Consistent with previous findings, the ASAQ efficacy was uniformly high (above 95%) in all three sites.

Recent reports of AL efficacy estimates below 90% from the Mikalayi site in the Democratic Republic of the Congo (DRC), less than 500 km from Lunda Sul and the closest efficacy monitoring site to Lunda Sul, provide additional context of potential warning signs for AL in the region. Moreover, the low day 28 AL efficacy in Lunda Sul in this study was observed in the backdrop of a relatively low rate of day 2 clearance, as only 68% of participants were microscopy negative on day 2, compared to 97% in the same site in 2017. Although day 3 slide negativity, the indicator used by the WHO to assess the ability of artemisinin to clear initial parasitemia, was 95% in this arm, the day 2 clearance rate is a notable finding. Finally, while the relatively short half-life of lumefantrine (14) is a likely explanation for the large number of reinfections in the AL arms, the large number of recrudescences in the AL Lunda Sul arm likely reflects underlying decreased susceptibility to lumefantrine in the parasite population.

There was no molecular evidence of artemisinin resistance, as all failure samples had wild-type *pfk13* sequences. The results of analyses of *pfcrt* and *pfmdr1* sequences were consistent with literature showing an overrepresentation of the 76T *pfcrt* allele in amodiaquine treatment failures and a predominance of the N86 *pfmdr1* allele in AL treatment failures. Interpretation of the latter finding should be done in the context of previous reports of the high population prevalence of the N86 allele in parasites circulating in Angola and the high prevalence of the N86 allele even in ASAQ treatment failures in this study. Molecular characterization of all day 0 samples, not just failures, will allow a better understanding of the population prevalence of these markers. Notably, fixation or near fixation of the N86 allele, together with the clinical evidence of reduced efficacy, might have implications for the future of AL use in Angola.

The unavailability of quality-controlled DP precluded an assessment of its efficacy in this round of monitoring in Angola. The recent introduction of new prequalified formulations should ensure that DP returns to being evaluated in future rounds. The inability to directly observe evening AL doses means that lumefantrine drug level measurement might be warranted, as in previous rounds (9), especially in the Lunda Sul site. Consecutive assessment of the two study drugs, as opposed to randomization, and small sample sizes do not allow direct comparison of drug efficacies. Moreover, because estimates of drug efficacies are close to 90%, substantially larger sample sizes will be required for a definitive determination of whether the true efficacy is above or below this threshold in future rounds. In the meantime, consideration of alternatives to AL in Angola may be warranted. In a larger context, our findings of consistent long-term trends of suboptimal efficacy of AL in Angola, together with recent results showing corrected AL efficacies substantially below 90% in West Africa (Adama Gansane, personal communication), suggest that the malaria control community might consider reducing the continent-wide overreliance on AL.

## MATERIALS AND METHODS

### Study design.

A prospective clinical outcome trial was conducted according to the standard 2009 WHO *in vivo* protocol (5). Two drugs, AL and ASAQ, were consecutively tested in each of the three sites for a total of six treatment groups. The three sentinel sites included M’Banza Congo in the hyperendemic Zaire Province in the north of the country, Saurimo in the hyperendemic Lunda Sul Province in the east, and the eponymous capital of the mesoendemic Benguela Province on the central western coast (see Fig. S1 in the supplemental material). The study was conducted from March to July 2019, coinciding with the high-malaria-transmission period.

### Study population.

Children with uncomplicated P. falciparum monoinfection presenting with febrile illness to sentinel site clinics were eligible for inclusion. Age and parasite density-based eligibility criteria reflected the different transmission levels, as dictated in the WHO protocol: 6 to 59 months of age and 2,000 to 200,000 parasites/μl in Zaire and Lunda Sul versus 6 to 143 months of age and 1,000 to 100,000 parasites/μl in Benguela. Children with signs of severe illness, antimalarial use in the preceding 2 weeks, allergy or hypersensitivity to the drugs, or hemoglobin levels of <8 g/dl were excluded from enrollment. A target of 100 participants per arm was chosen to provide a precision of 5% assuming a 25% combined exclusion and loss-to-follow-up rate and 95% efficacy.

### Study procedures.

Children were treated with either AL (Cipla, Mumbai, India) or ASAQ (Sanofi-Aventis, Maphar, Morocco) for 3 days. The dosage was weight based according to the manufacturers’ recommendations. All ASAQ doses were directly observed by study staff and were administered with water or juice. For AL, morning doses were observed by study staff and were administered with yogurt or milk. Parents or guardians were given the evening dose and a yogurt or milk packet to give at home. Study staff telephoned parents or guardians in the evening to remind them to administer the evening dose, and compliance was further assessed by requesting parents or guardians to bring empty blister packets to the clinic the following day.

Children were monitored daily for the first 4 days and then weekly thereafter for a total of 28 days. At each visit, study staff performed clinical exams, performed blood smears (except for day 1), and collected blood on Whatman 903 filter paper (GE Healthcare Life Sciences, Marlborough, MA, USA). Hemoglobin was assessed fortnightly using HemoCue Hb301 (Hemocue, Ängelholm, Sweden) or DiaSpect (EKF Diagnostics, Barleben, Germany) hemoglobinometers.

### Laboratory analysis.

Blood smear microscopy was done using thick and thin smears (5) by two microscopists. Discrepancies of more than 20% between parasite density estimates were resolved by a reading by a third microscopist. Monthly supervision visits included quality control of a randomly selected 10% of slides.

Dried blood spots were dried overnight and then placed in individual bags with a desiccant. After the end of the study, DNA was extracted using QIAamp DNA minikits (Qiagen, Hilden, Germany) at National Institute of Health laboratories in Angola. Extracted DNA was transported to U.S. Centers for Disease Control and Prevention (CDC) Malaria Branch laboratories in Atlanta, GA. Molecular analysis was conducted by visiting Angolan study investigators under the President’s Malaria Initiative-supported Antimalarial Resistance Monitoring in Africa Network capacity-building program (15). Paired day 0 and day-of-failure samples from cases of recurrent parasitemia and 60 randomly selected day 0 nonfailure samples were analyzed for seven microsatellite markers (TA1, poly-α, PfPK2, TA109, TA2490, C2M34, and C3M69) (16, 17). Following photo-induced electron transfer (PET)-PCR (18) confirmation of the presence of P. falciparum, fragment lengths of the microsatellite markers were measured using an ABI3130 xl genetic analyzer (Applied Biosystems, Foster City, CA) capillary sequencer and GeneMapper software (Applied Biosystems, Foster City, CA). Day 0 samples from early treatment failures (before day 7) and day 0 and day-of-failure samples from cases of recurrent parasitemia (on or after day 7) were analyzed using a targeted deep-amplicon-sequencing workflow that included the full *pfk13*, *pfcrt*, and *pfmdr1* genes (19).

### Statistical analysis.

Data were double entered into an Excel database (Microsoft, Redmond, WA). Baseline characteristics were summarized by treatment group. The proportions of participants with negative slides on day 2 and day 3 were calculated. Individuals finishing follow-up were classified as early treatment failures, cases of recurrent parasitemia, or cases of adequate clinical and parasitological response (ACPR) according to standard WHO definitions (5). For molecular correction, microsatellite genotyping data from day 0 and day-of-failure samples from cases of recurrent parasitemia were compared using a Bayesian algorithm, and the posterior probability of recrudescence was calculated based on the observed allele data (20, 21). For tabulation, recrudescences were defined when the posterior probability of recrudescence was >0.5. The corrected efficacy and corresponding 95% confidence intervals (CIs) were calculated using the Kaplan-Meier estimate of the survival function at day 28 and the posterior sample of calculated probabilities of recrudescence (20).

### Ethical review.

The study was reviewed by the human subjects review boards at the Angolan Ministry of Health, and the activity was approved as a nonresearch program evaluation by the Office of the Associate Director for Science at the CDC’s Center for Global Health (protocol 2014-233c). Parents or guardians of study participants provided written informed consent.

### Data availability.

The full clinical data set has been uploaded to the WorldWide Antimalarial Resistance Network and WHO repositories. Full microsatellite genotyping data are available in Tables S1 and S2 in the supplemental material.

## Supplementary Material

Supplemental file 1
