# Supplementary material for: Continued Low Efficacy of Artemether-Lumefantrine in Angola in 2019
Source: Antimicrob Agents Chemother. 2021 Jan 20;65(2):e01949-20. doi: 10.1128/AAC.01949-20 (PMC7849008; doi:10.1128/AAC.01949-20)
Supplement: Supplemental file 1 [file AAC.01949-20-s0001.pdf]

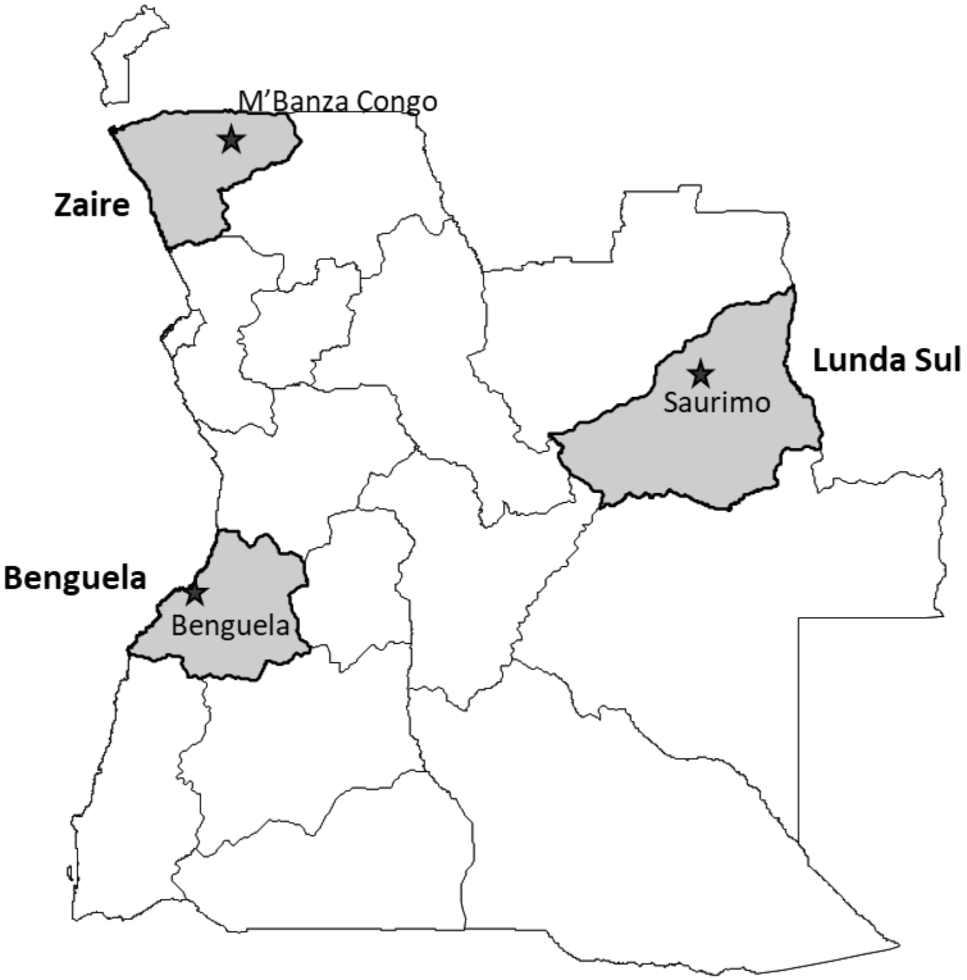


**Supplementary Figure S1**. Location of fixed sentinel sites for therapeutic efficacy monitoring in Angola, 2019

**Supplementary Table S1**: Microsatellite lengths of paired Day 0 - Day of Failure samples from cases of recurrent parasitemia observed during 2019 round of therapeutic efficacy monitoring in Angola. Last column shows posterior probability of recrudescence obtained from Bayesian analysis of observed data and reflects the statistical support that the recurrent parasitemia is due to a recrudescence (posterior probability = 1) or reinfection (posterior probability = 0).

| **Chromosome** | **2** | **3** | **6** | **4** | **12** | **10** | **6** | Posterior Probability of Recrudescence |
|---|---|---|---|---|---|---|---|---|
| **Marker Name** | **313 C2** | **383 C3** | **TA1** | **POLYA** | **PFPK2** | **2490** | **TA109** | |
| **Sample ID** | | | | | | | | |
| BD19009D0 | 228 | 123 139 | 171 | 153 | 168 186 | 81 84 | 175 | |
| BD19009D28 | 216 | 123 145 | 168 177 | 150 168 | 168 | 84 | 175 199 | 0.27 |
| BD19010D0 | 218 | 137 | 177 | 168 | 165 | 84 | 172 | |
| BD19010D28 | 218 228 | 123 | 165 180 | 150 168 177 | 162 168 | 84 | 172 184 | 0.35 |
| BD19016D0 | 228 | 123 | 165 | 171 | 162 | 81 | 160 | |
| BD19016D21 | 244 | 145 157 | 174 | 135 165 | 162 171 | 81 84 | 178 199 | 0.00 |
| BD19017D0 | 220 | 145 | 159 | 162 | 162 | 84 | 163 | |
| BD19017D21 | 246 | 145 | 165 | 162 | 171 186 | 84 | 175 199 | 0.00 |
| BD19020D0 | 252 | 145 | 171 | 150 | 162 | 84 | 163 | |
| BD19020D28 | 230 | 137 | 162 171 | 168 | 174 201 | 81 | 163 | 0.00 |
| BD19026D0 | 244 | 171 | 189 | 165 | 189 | 81 | 160 | |
| BD19026D28 | 238 | 145 | 168 | 159 | 180 | 84 | 175 | 0.00 |
| BD19031D0 | 220 264 | 123 149 | 159 177 | 141 159 | 168 177 | 81 84 | 172 184 | |
| BD19031D28 | 216 | 139 | 165 171 | 144 165 | 165 168 | 81 84 | 178 | 0.00 |
| BD19042D0 | 236 | 123 | 177 180 | 156 | 180 186 | 81 84 | 175 | |
| BD19042D21 | NA | NA | 189 | 162 | 162 204 | 84 | 163 199 | 0.00 |
| BD19065D0 | 220 | 137 | 204 | 156 | 195 | 84 | 172 | |
| BD19065D28 | 230 252 | 137 | 162 168 174 | 153 168 | 162 186 | 84 | 160 | 0.00 |
| BD19066D0 | 226 | 145 | 204 | 168 | 171 | 84 | 163 | |
| BD19066D14 | 226 | 145 | 204 | 168 | 171 | 84 | 163 199 | 1.00 |
| BD19094D0 | 216 | 147 | 171 | 153 | 165 | 84 | 178 | |
| BD19094D21 | 216 | 147 | 171 | 153 | 165 | 84 | 178 | 1.00 |
| ZL19201D0 | 232 | 123 | 168 | 159 | 180 | 84 | 175 | |
| ZL19201D21 | 236 | 143 | 183 | 150 | 174 | 72 | 175 | 0.00 |
| ZL19203D0 | 238 | 137 | 162 | NA | 162 | 84 | 172 | |
| ZL19203D14 | 242 | 125 | 165 168 | 138 180 | 159 192 | 81 84 | 160 172 199 | 0.00 |
| ZL19204D0 | 250 | 143 | 168 | 129 | 165 180 | 81 | 172 | |
| ZL19204D21 | 220 | 123 | 195 | 153 171 | 180 | 81 | 175 | 0.64 |
| ZL19209D0 | 240 | 143 | 159 | 156 | 183 | 84 | 175 | 1.00 |

| | | | | | | | | |
|---|---|---|---|---|---|---|---|---|
| ZL19209D21 | 240 | 143 | 159 | 156 | 183 | 84 | 175 | |
| ZL19218D0 | 262 | 141 | 168 | 156 | 186 | 84 | 190 | |
| ZL19218D28 | 234 | 123 | 174 | 144 | 174 | 84 | 160 | 0.00 |
| ZL19231D0 | 220 248 | 123 151 | 168 | 153 165 | 159 | 84 93 | 160 | |
| ZL19231D28 | 218 224 248 | NA | 165 | 156 | NA | 84 | 199 | 0.81 |
| ZL19235D0 | 214 252 | 165 | 162 180 | 138 | 171 | 81 | 175 | |
| ZL19235D28 | 218 | 137 | 168 | 150 | 159 | 87 | 163 | 0.00 |
| ZL19236D0 | 226 | 125 139 | 174 | 150 | 168 | 81 84 | 160 | |
| ZL19236D14 | 226 | 139 | 174 | 150 | 168 | 84 | 160 199 | 1.00 |
| ZL19244D0 | 238 | 147 | 159 | 147 | 174 | 84 | 163 | |
| ZL19244D21 | 238 | 147 | 159 | 147 | 174 | 84 | 163 | 1.00 |
| ZL19246D0 | 236 | 139 | 159 | 165 | 165 | 84 | 172 | |
| ZL19246D21 | 234 | 123 | 174 | 153 | 174 | 81 | 172 | 0.00 |
| ZL19251D0 | 218 224 240 | 123 139 | 159 165 | 171 | 165 186 192 | 84 | 175 | |
| ZL19251D28 | 240 | 139 | 159 | 177 | 159 | 84 | 175 184 199 | 0.90 |
| ZL19260D0 | 238 | 125 | 186 | 150 | 165 186 | 84 | 175 | |
| ZL19260D21 | 212 | 125 | 177 | 180 | 159 | 72 84 | 160 | 0.69 |
| ZL19262D0 | 242 | 125 | 162 192 | 138 177 | 168 186 | 84 | 172 | |
| ZL19262D28 | 220 | 125 | 168 | 141 | 165 | 81 84 | 163 | 0.26 |
| ZL19274D0 | 224 | 123 | 174 | 141 147 | 171 183 192 | 81 84 | 160 | |
| ZL19274D21 | 224 | 123 153 | 174 | 141 147 177 | 171 183 | 81 84 | 160 | 1.00 |
| ZL19293D0 | 248 | 139 | 162 | 153 | 165 | 84 | 181 | |
| ZL19293D29 | 248 | 141 | 162 | 153 | 165 | 84 | 160 | 0.00 |
| ZL19294D0 | 252 | 157 | 174 | 153 | 174 | 84 | 160 | |
| ZL19294D28 | 236 | 135 | 138 | 168 | 165 | 84 | 184 | 0.00 |
| LL19000D0 | 244 | 123 | 192 | 114 | 165 | 84 | 160 | |
| LL19000D21 | 244 | 123 | 192 | 114 | 165 | 84 | 160 | 1.00 |
| LL19007D0 | 244 | 165 | 174 | 141 | 189 | 81 | 172 | |
| LL19007D28 | 244 | 165 | 174 | 141 | 189 | 81 | 172 | 1.00 |
| LL19013D0 | 246 | 141 | 174 | 141 | 165 | 84 | 163 | |
| LL19013D21 | 246 | 141 | 174 | 141 | 165 | 84 | 163 | 1.00 |
| LL19014D0 | 230 | 123 | 183 | 171 | 168 | 72 | 175 | |
| LL19014D21 | 230 | 123 | 183 | 171 | 168 | 72 | 175 | 1.00 |
| LL19016D0 | 226 | 123 | 180 | 150 | 165 | 87 | 199 | |
| LL19016D14 | 228 | 141 149 | 186 | 150 171 | 156 171 | 84 | 160 199 | 0.00 |
| LL19017D0 | 240 | 123 | 174 177 | 150 | 162 174 | 84 | 160 184 | |
| LL19017D21 | 240 | 123 | 174 | 150 | 174 | 84 | 160 | 1.00 |

| | | | | | | | | |
|---|---|---|---|---|---|---|---|---|
| LL19033D0 | 228 | 169 | 159 | 144 | 183 | 84 | 172 | |
| LL19033D21 | 228 | 169 | 159 | 144 | 177 183 | 84 | 172 | 1.00 |
| LL19054D0 | 238 | 123 135 | 168 171 | 150 156 | 162 174 | 84 | 160 175 | |
| LL19054D21 | 220 228 234 260 | 125 | 168 171 | 153 | 162 171 174 | 81 84 | 160 175 | 0.80 |
| LL19062D0 | 238 | 149 | 165 | 171 | 162 | 84 | 172 | |
| LL19062D14 | 244 | 139 | 171 | 147 156 | 168 | 84 | 175 199 | 0.00 |
| LL19071D0 | 274 | 149 | 165 | 162 | 177 | 72 | 160 | |
| LL19071D21 | 274 | 149 | 165 | 162 | 177 | 75 | 160 | 0.99 |
| LL19073D0 | 236 | 123 | 171 | 150 | 162 174 | 81 84 | 172 | |
| LL19073D21 | 236 | 123 | 171 | 150 | 162 | 84 | 172 | 1.00 |
| LL19081D0 | 226 232 | 123 135 145 | 162 165 168 | 153 162 177 | 165 180 | 84 | 172 | |
| LL19081D21 | 226 | 145 | 168 | 177 | 180 | 84 | 172 | 1.00 |
| LL19089D0 | 256 | 137 | 177 | 150 | 159 177 | 84 | 163 | |
| LL19089D14 | NA | 137 | 165 | 144 | 159 171 177 | 84 | 172 184 199 | 0.82 |
| LL19091D0 | 214 | 123 145 | 159 171 | 147 156 171 | 162 171 | 81 84 | 175 190 | |
| LL19091D14 | 232 | 123 | 159 | 147 | 171 | NA | 175 184 199 | 0.88 |
| ZD19017D0 | 238 | 165 | 195 | 144 | 189 | 81 | 184 | |
| ZD19017D28 | 238 | 165 | 195 | 144 | 189 | 81 | 184 | 1.00 |
| ZD19028D0 | 244 | 141 | 162 | 156 171 | 171 | 84 | 172 | |
| ZD19028D21 | 250 | 135 | 159 | 156 | 165 | 84 | 160 | 0.00 |
| ZD19031D0 | 258 | 139 | 162 | 171 | 189 | 84 | 163 | |
| ZD19031D21 | 238 | 139 | 171 | 156 | 168 | 84 | 175 | 0.00 |
| ZD19039D0 | 236 ` | 145 | 162 | 153 | 186 | 84 | 172 | |
| ZD19039D14 | 212 242 | 139 147 | 159 168 | 150 159 | 168 | 84 | 160 175 | 0.00 |
| ZD19043D0 | 224 | 139 | 168 183 | 132 138 | 159 168 186 | 84 90 | 160 | |
| ZD19043D28 | 224 | 133 | 168 180 | 168 | 186 | 84 | 172 184 | 0.74 |
| ZD19047D0 | 254 | 147 | 195 | 168 | 174 | 84 | 160 | |
| ZD19047D28 | 226 | 149 | 171 | 150 | 165 | 84 | 172 | 0.00 |
| ZD19066D0 | 234 250 | 123 133 | 168 | 150 153 162 | 162 177 189 | 84 | 172 184 | |
| ZD19066D21 | 258 | 135 139 | 141 168 | 153 162 | 162 | 81 84 | 172 | 0.86 |
| ZD19072D0 | 234 | 139 | 177 | 150 | 165 | 84 | 160 | |
| ZD19072D28 | 218 | 123 | 162 | 150 | 180 | 81 | 178 187 | 0.00 |
| ZD19075D0 | 226 | 145 | 165 | 150 | 156 | 81 | 172 | |
| ZD19075D21 | 252 | 123 | 168 | 153 | 165 | 84 | 175 | 0.00 |
| ZD19081D0 | 222 | 147 | 171 183 | 153 | 168 | 81 84 | 172 | |
| ZD19081D21 | 224 238 | 145 153 | 165 168 | 132 150 177 | 162 168 189 | 81 84 | 172 | 0.25 |
| ZD19092D0 | 242 | 123 | 177 | 150 | 165 | 84 | 163 | 0.00 |

| | | | | | | | | |
|---|---|---|---|---|---|---|---|---|
| ZD19092D28 | 242 | 123 | 168 174 | 141 150 | 168 183 | 84 | 166 175 | |
| ZD19098D0 | 236 | 151 | 174 | 150 | 159 | 84 | 160 | |
| ZD19098D21 | 238 | 139 | 159 183 | 150 162 165 | 159 192 | 81 84 | 172 | 0.06 |
| ZD19100D0 | 252 | 139 | 159 | 159 | 186 | 84 | 160 | |
| ZD19100D28 | 250 | 123 | 174 | 165 | 186 | 87 | 160 | 0.00 |

**Supplementary Table S2**: Diversity characterized using Simpson's index for neutral microsatellite loci observed during 2019 round of therapeutic efficacy monitoring in Angola.

| Microsatellite marker | Site | | |
|---|---|---|---|
| | Zaire | Benguela | Lunda Sul |
| 313 C2 | 0.062 (0.059–0.065) | 0.074 (0.069–0.078) | 0.070 (0.065–0.074) |
| 383 C3 | 0.122 (0.110–0.131) | 0.174 (0.157–0.187) | 0.151 (0.129–0.168) |
| TA1 | 0.099 (0.092–0.104) | 0.119 (0.111–0.125) | 0.114 (0.105–0.122) |
| POLYA | 0.086 (0.080–0.091) | 0.107 (0.100–0.113) | 0.098 (0.088–0.105) |
| PFPK2 | 0.105 (0.098–0.110) | 0.121 (0.110–0.130) | 0.120 (0.111–0.128) |
| 2490 | 0.381 (0.354–0.406) | 0.572 (0.523–0.618) | 0.382 (0.338–0.422) |
| TA109 | 0.159 (0.148–0.169) | 0.173 (0.163–0.180) | 0.158 (0.147–0.167) |

Numbers in parentheses represent 95% credible intervals. Simpson's index is defined as $\sum f_i^2$, where $f_i$ is the frequency of the *i*th allele. The index varies from 0 (highest diversity) to 1 (lowest diversity).
